# Supplementary material for: Neu3 Sialidase Activates the RISK Cardioprotective Signaling Pathway during Ischemia and Reperfusion Injury (IRI)
Source: Int J Mol Sci. 2022 May 29;23(11):6090. doi: 10.3390/ijms23116090 (PMC9181429; doi:10.3390/ijms23116090)
Supplement: Supplementary file 1 [file ijms-23-06090-s001.zip › ijms-1624534-supplementary.pdf]

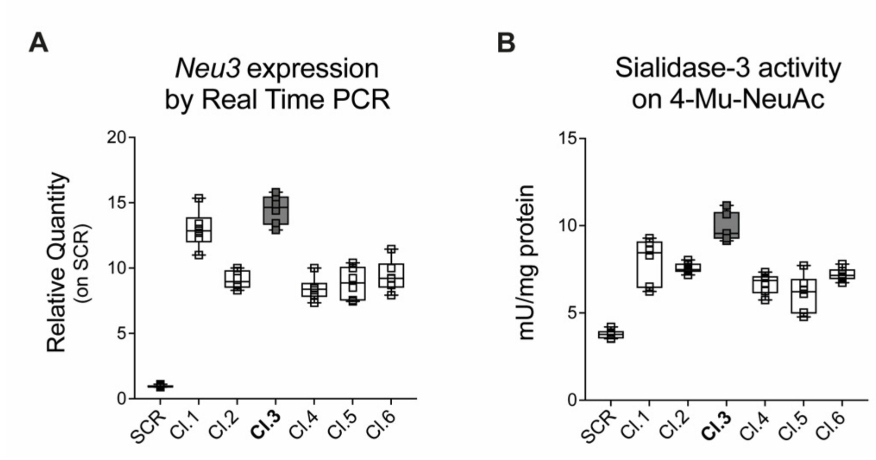

Figure S1: H9c2 *Neu3* overexpressing clones' selection by *Neu3* expression (A) and sialidase-3 activity analysis (B). Each square in the graphs represent an experimental replicate.

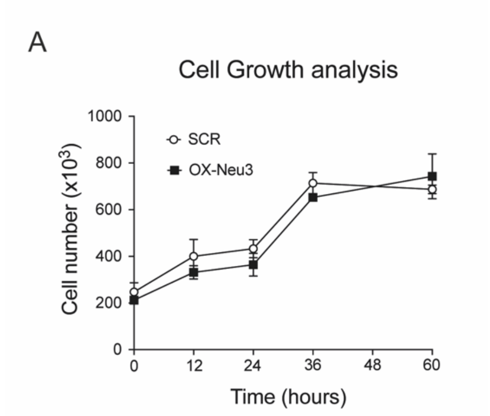

Figure S2: Cell growth analysis of SCR and OX-Neu3 H9c2 cells in normal culturing conditions.

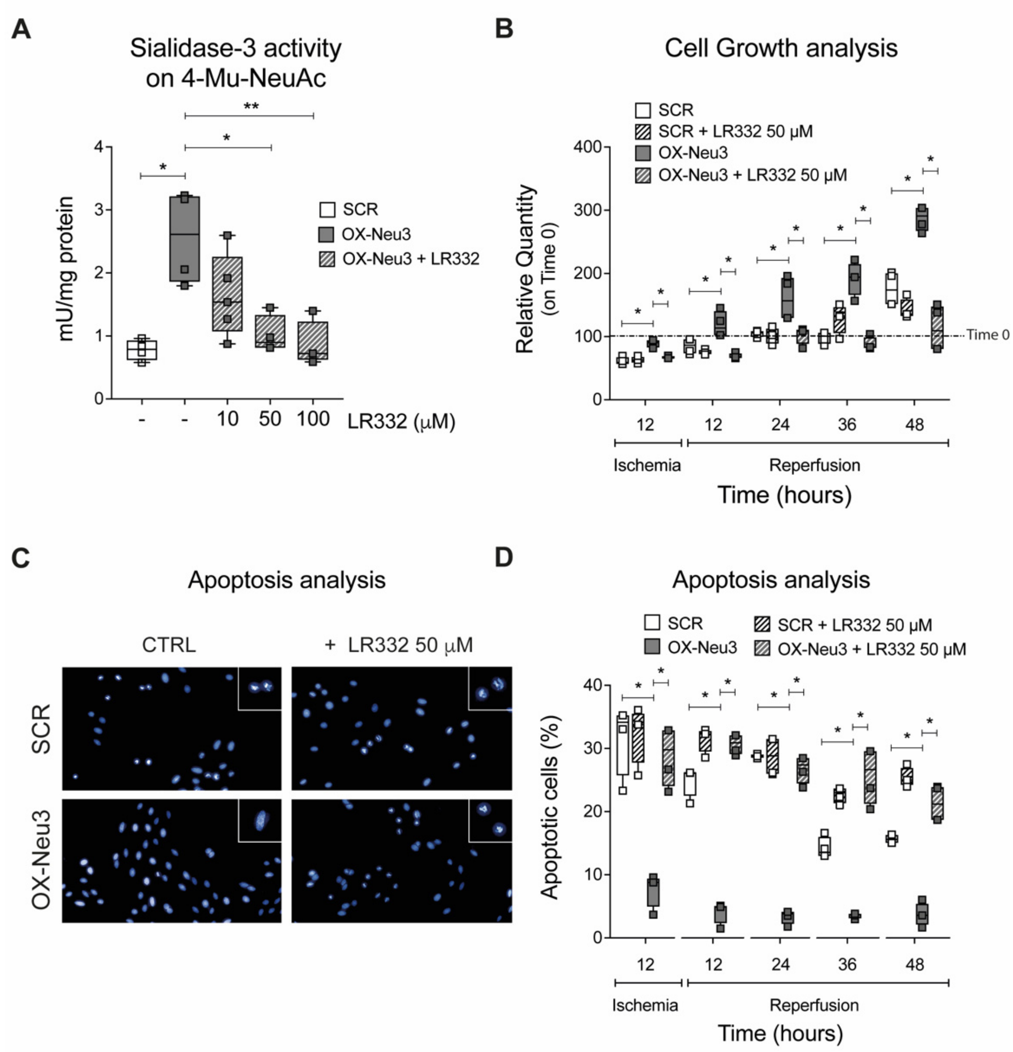

Figure S3. Effects of sialidase-3 inhibition by LR332 in H9c2 cells exposed to IRI. (A) Sialidase-3 activity analysis in the presence of different LR332 concentrations (10-50-100 μM); (B) Cell growth analysis; (C) apoptotic nuclei staining by Hoechst 33342; (D) apoptosis level analysis expressed as percentage of apoptotic nuclei per total nuclei. Each square in the graphs represent an experimental replicate. Statistical significance was determined by the nonparametric Kruskal-Wallis test. *P<0.05; **P<0.01.

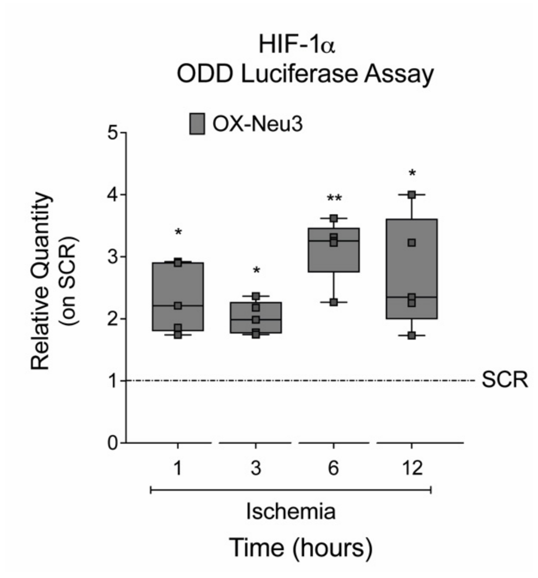

Figure S4: HIF-1α stabilization during ischemia in OX-Neu3 H9c2 cells. Data are expressed as relative quantity compared with scramble cells, used as internal control for each time point analyzed (*SCR dashed line*). Statistical significance was determined by the nonparametric Kruskal-Wallis test. *P<0.05; **P<0.01.

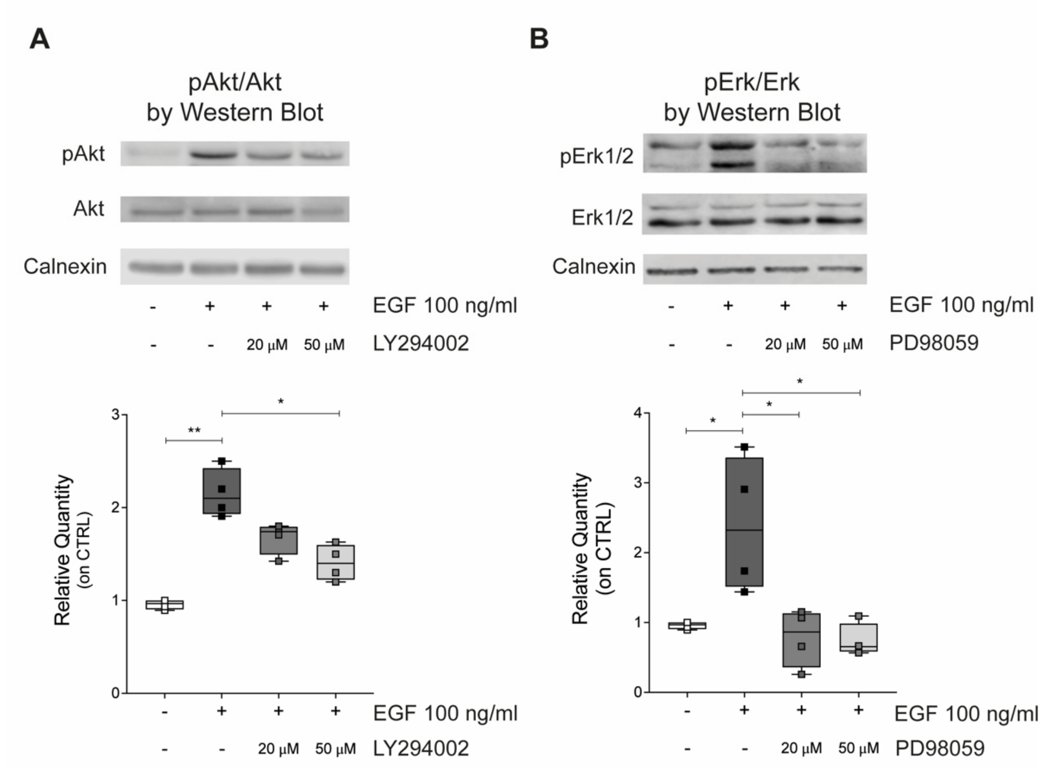

Figure S5: Evaluation by Western Blot of the best concentration of the RISK pathway inhibitors LY294002 and PD98059. The graphs represent the densitometric analysis of the WB bands (lower panels). Each square in the graphs represent an experimental replicate. Statistical significance was determined by the nonparametric Kruskal-Wallis test. *P<0.05; **P<0.01.
